# Severe Affection of the Stomach and Duodenum, Imitating Scirrhus of the Pylorus

**Published:** 1829-01-01

**Authors:** 


### XXXIV.

### Severe Affection of the Stomach and Duodenum. By M. Bricheteau.

*Case.* A gentleman, (Mr. M.) aged 65

years, of strong constitution, bilious temperament, and who had enjoyed good health, having led a sober and active life, was the subject of severe misfortunes and moral afflictions in the year 1825, which made a great impression on body as well as mind. He became affected with bad digestion, experiencing pain and weight in his stomach, after eating. On the 11th of April, 1828, M. Bricheteau was consulted. The patient then complained of rather severe pain in the umbilical region, which was tense and somewhat shining — his appetite was gone — his bowels obstinately confined; but he had neither fever nor heat of skin. Twenty leeches were applied to the anus—emollient fomentations to the abdomen—tepid bath—diluent drinks — lavements, &c. These means having relieved the pain, the bowels were opened by means of castor oil, and the patient was soon able to take solid food. On the 19th of the same month, late at night, M. Bricheteau was again summoned to the patient, who, for six hours previously, had been in racking pain, in the abdomen, which was now so tense and tender, that no examination could be made. The cries of the patient were most distressing, though he was naturally courageous and placable. Yet there was no febrile heat nor acceleration of pulse. Warm opiate frictions, and an opiate internally, quieted the intensity of the symptoms; but it was thought advisable, next day, to apply 20 leeches to the right hypochondrium, and renew the fomentations, &c. The abdomen being rendered softer, M. Bricheteau made an examination, and felt distinctly an oblong tumour or hardness in the region of the pylorus. There was still some pain in the part, and there was now some febrile action in the pulse. The most rigorous diet was prescribed, and cataplasms, with laudanum, were kept to the epigastrium. The pain became more and more obtuse, but the patient emaciated—his strength declined—he had eructations—obstinate constipation—no sleep—and finally, after a few days of great malaise about the region of the stomach, he vomited up a quantity of black stuff resembling coffee-grounds. The patient felt relieved by this evacuation, but was not improved in other respects. In eight days he had a repetition of the black-vomiting, and now got so low that he made his will, and considered himself a dying man. So did his physician. Nevertheless the Doctor recommended repeated frictions, with the antimoniated ointment over the umbilical and epigastric regions. It required eight days to bring out a plentiful crop of pustules, so dry and harsh was the skin. The vomiting ceased, but the oval tumour could still be felt, and was painful on pressure. The constipation also continued. After ten days immunity from sickness, the vomiting of black matters was renewed by the attempt to take some asparagus. From this time he kept on a system of the most rigid abstinence, with Seltzer-water for drink, and the tumour gradually lessened till it entirely disappeared. Ultimately he was able to resume light animal and vegetable food—his bowels became more free, and his health was re-established.

Dr. Bricheteau apprehended, for a considerable time, that the disease was an organic affection of the pylorus. The result has convinced him, that it must have been an inflammatory engorgement of that part, not a scirrhous induration. He attaches great importance to the Seltzer-water, antimoniated ointment, and abstinence. To the two last we give full credit, but how far the Seltzer-water contributed to the cure, we will not pretend to say. The case is interesting, as shewing that we should not too hastily pronounce a disease to be scirrhus of the pylorus, though attended with all the usual symptoms of that dreadful disease.—*Journ. Comp.*

---

